# TTX‐S/TTX‐R Na^+^ Currents Coordinately Fine‐Tune Depolarization/Firing Capability Revealed by Voltage Derivatives/Displacement Current Phase Plots With Dynamic Current‐Clamp Simulation in Rat Visceral Sensory Neurons

**DOI:** 10.1111/apha.70239

**Published:** 2026-05-03

**Authors:** Zhang Jing‐ran, Fu Hui‐xiao, Li Xing‐yu, Zhang Hong‐fei, Dou Tian‐min, Li Bai‐yan, Wu Di

**Affiliations:** ^1^ Department of Pharmacy The 2nd Affiliated Hospital of Dalian Medical University Dalian China; ^2^ Department of Pharmacology, School of Pharmacy Harbin Medical University Harbin China

**Keywords:** action potential, depolarization, dynamic‐current clamp, ion channels, neuronal encoding, phase plot, repolarization, tetrodotoxin

## Abstract

**Aim:**

To investigate how tetrodotoxin‐sensitive (TTX‐S) and tetrodotoxin‐resistant (TTX‐R) Na^+^ channels coordinately fine‐tune action potential (AP) depolarization and firing capability in rat nodose visceral sensory neurons.

**Methods:**

APs were recorded by ruptured‐patch current clamp in unmyelinated C‐type and myelinated Ah‐type neurons from isolated and sliced nodose ganglia. Voltage derivatives and displacement current phase plots were used to determine the kick‐in voltage of TTX‐R following TTX‐S activation. Myelinated A‐type neurons, which express TTX‐S exclusively, served as a model for dynamic current‐clamp (DCC) simulation, in which gNa0 (TTX‐S) and gNa1 (TTX‐R) were injected separately or in combination.

**Results:**

Voltage derivatives and phase plots revealed a biphasic upstroke in C‐ and Ah‐type neurons, indicating sequential TTX‐S then TTX‐R activation. The TTX‐R kick‐in voltage was more negative in Ah‐type than in C‐type neurons and was strongly inversely correlated with the maximal upstroke velocity. DCC faithfully reproduced both AP types; TTX‐R reactivation generated the C‐type repolarization hump, and AP peak was preserved through proportional gNa0/gNa1 compensation. Increasing the integrated step size of gNa1 delayed TTX‐R recruitment, reduced the second Na^+^ peak, and progressively impaired repetitive firing, whereas the TTX‐S peak remained unchanged.

**Conclusion:**

TTX‐S and TTX‐R Na^+^ channels coordinate AP depolarization sequentially and compensatorily: TTX‐S initiates the upstroke, whereas TTX‐R is recruited later and reactivates during repolarization to constrain firing frequency. Combining patch‐clamp with DCC simulation provides novel insight into visceral sensory neuron excitability.

## 1 Introduction

It is well documented that tetrodotoxin‐sensitive (TTX‐S) [1, 2, 3] and TTX‐resistant (TTX‐R) sodium (Na^+^) channels [4, 5, 6] are co‐expressed in myelinated Ah‐ and unmyelinated C‐type visceral afferent neurons housed in the nodose ganglia (NG) and contribute to the afferent reflex functions under physiological [7, 8] and disease conditions [9, 10]. However, it is largely unknown how these two channel classes coordinate in the depolarization process of action potential (AP) and firing capability.

In general, the patch‐clamp technique is a unique tool for an ion channel study, but the TTX‐S component is essential for initial depolarization, and AP depolarization can hardly be elicited in the presence of TTX, even if both categories of NG neurons functionally express TTX‐R, which is an obvious technical limitation to reach our goal. To overcome this limitation, we employed myelinated A‐type NG neurons from isolated and sliced preparations as our study model. These neurons exclusively express TTX‐S channels that can be completely blocked by TTX, providing an ideal platform for controlled channel substitution experiments. We then utilized dynamic current‐clamp (DCC) [11, 12, 13, 14], a powerful computational technique that allows real‐time injection of simulated conductances to mimic specific ion channel activities [12, 14]. This approach has been successfully applied to investigate ionic mechanisms underlying neuronal excitability in various preparations [11, 13, 15, 16]. Using DCC, digitized conductances gNa0 and gNa1 (representing TTX‐S and TTX‐R components, respectively) could be injected separately or in combination to simulate AP and repetitive discharge characteristics. These combined techniques allow us to distinguish the current dynamics of each component and their coordination during AP depolarization.

In the present study, we combine experimental approaches with dynamic current‐clamp (DCC) modeling to address two fundamental questions regarding the dynamic regulation of Na^+^ currents in nodose sensory neurons: (1) Are distinct classes of Na^+^ currents (TTX‐S and TTX‐R) expressed in the same neurons all required for action potential (AP) depolarization and neuronal excitability, or do they play distinct sequential roles? (2) How do TTX‐S and TTX‐R components interact during AP development, and can their dynamic interactions be quantitatively characterized through DCC simulation? To address these questions, we employ three complementary approaches: displacement current phase plot analysis to identify TTX‐R recruitment during native APs, voltage derivative analysis to reveal the sequential activation of channel subtypes, and DCC modeling using digitized conductance inputs (gNa0 representing TTX‐S/mostly Nav1.7 and gNa1 representing TTX‐R/mostly Nav1.8) to simulate and manipulate individual Na^+^ channel contributions. By systematically varying gNa0 and gNa1 inputs, we aim to determine the precise voltage at which TTX‐R channels are recruited (kick‐in voltage), characterize the compensatory relationship between channel subtypes, and elucidate how their coordinated activation maintains neuronal excitability. This integrated approach provides unprecedented insight into the dynamic regulation of Na^+^ currents underlying visceral sensory neuron function.

## 2 Materials and Methods

### 2.1 Animals

Adult female Sprague–Dawley (SD) rats (180 ± 20 g) were purchased from Weitong‐Lihua Experimental Animal Tech Corp (Grade: SPF, Certificate #: SCXK, 2012‐0001, Beijing, China) and housed in a pathogen‐free environment under controlled temperature and humidity with a 12‐h light cycle and free access to rodent food and tapped water at the animal facility at Harbin Medical University. All rats were kept in the above environment for at least 1 week before use. All animal protocols were pre‐approved by the Institutional Animal Care and Use Committee of the School of Pharmacy at Harbin Medical University, which are in accordance with the recommendations of the Panel on Euthanasia of the American Veterinary Medical Association and the National Institutes of Health publication “Guide for the Care and Use of Laboratory Animals” (http://www.nap.edu/readingroom/books/labrats/).

### 2.2 Chemicals

Tetrodotoxin (TTX, #1078) was ordered from Tocris (Ellisville, MO, USA). All other chemicals and enzymes for isolated or slice preparation as well as those for recording solutions were obtained from standard routine commercial sources.

### 2.3 Neuron Preparation

After complete relaxation with CO_2_ in an anesthesia induction chamber following the administration of a cocktail of ketamine/xylazine (0.1 mL/100 g, i.p.), the rats were immediately sectioned at the mid‐axillary region to ensure sufficient length vagus nerve and nodose ganglia (NG) of both sides were dissected quickly with caution upon the procedures [17, 18]. For isolated neurons preparation, the ganglia were washed for 3 times with ice‐cold Hank's Balanced Salt (Life Tech, Frederick, MD, USA) and digested using Papain (10 units/mL) for 20 min at 37°C, followed by a digestion cocktail containing 1.0 mg/mL Collagenase type‐II (Worthington Biochem. Corp., Lakewood, NJ, USA) and 2.5 mg/mL Dispase (Roche, USA) for additional 30 min at 37°C. The ganglion tissue was then washed twice in Dulbecco's Modified Eagle Medium (HyClone, Logan, UT, USA), re‐suspended in DMEM with 10% Fetal Bovine Serum (HyClone), 10% heat‐inactivated horse serum (Invitrogen, Grand Island, NY, USA), and 1% L‐glutamine (Sigma, St Louis, MO, USA), and gently triturated with an aspiration pipette. The resulting cell suspension was placed onto poly‐D‐lysine (Sigma, St Louis, MO, USA) coated coverslips (10 × 10 mm diameter, Bellco Glass Inc., Vineland, NJ, USA) and incubated for at least 4 h at 37°C in a standard culture environment before patch recording.

### 2.4 Nodose‐Slice Preparation

NG slices were prepared according exactly to our procedures described previously [17]. Briefly, after complete relaxation, the left vagus nerve (> 2 cm in total length) was carefully dissected and the bulbous section of the NG was then trimmed under a stereomicroscope to open the connective membrane without damaging the afferent fiber. The NG with the entire vagus nerve was then treated with the enzyme solutions mentioned above and placed in the recording chamber for at least 1 h before recordings.

### 2.5 Afferent Fiber Type Classification

For isolated neurons, myelinated Ah‐ and unmyelinated C‐types were determined by a set of waveform parameters [18] such as action potential (AP) firing threshold (APFT), AP duration (APD_50_), maximal up‐stroke (UV_MAX_), and down‐stroke (DV_MAX_) velocity and repolarization hump. For slice preparation, afferent fiber conduction velocity (CV) was measured by dividing the vagus nerve length between the two electrodes by the AP propagation time. At room temperature, the CV was 1.0 m/s for C‐types and ≥ 2 m/s for Ah‐types [18].

### 2.6 Electrophysiology

All experiments were conducted using the ruptured patch technique and Axoclamp 700A amplifier (Axon Instruments, Scottsdale, AZ, USA). For all recordings, patch pipettes (#7052, Corning, NY, USA) were pulled (P‐87; Sutter, Novato, CA, USA) and polished (MF‐830; Narishige, Tokyo, Japan) down to a resistance of 1–1.5 MΩ. Following the formation of a gigaseal, the pipette capacitance was compensated. Current clamp mode was used for recording somatic AP discharge. Details regarding holding potentials and voltage step protocols are presented along with the data in Section 3. All data were low‐pass filtered to 10 kHz and digitized at the rate of 40 kHz. The experimental protocols and data collection were carried out using the Digidata 1440A under the control of pCLAMP 10 (Axon Instruments) operating on a PC platform.

### 2.7 Recording Protocols

For the current‐clamp recordings, three stimulus protocols were used to evoke somatic APs from resting membrane potential (RMP). First, a brief constant pulse (Figure S1) of sufficient magnitude (≤ 500 μs, ≥ 400 pA for the 1st pulse) to elicit a single AP was applied through the patch electrode. Second, step current (500–1000 ms, ≤ 200 pA for the 1st step) was applied to assess the repetitive discharge (Figure S2). Third, a monophasic constant current pulse was applied to the vagus nerve using a platinum (90%)/iridium (10%) bipolar electrode (2 mm spacing of 3 mm uninsulated tips) (Figure S3). The pulse duration was individually adjusted for each slice preparation (typically 0.5–2.0 ms) to account for variations in nerve length, diameter, and surrounding connective tissue between animals, whereas maintaining stimulus intensity at safe levels to avoid nerve damage. The cathode was positioned a measured distance (~1–1.5 cm) from the center of the NG, which was adjusted by the factor of ~0.7 since the nerve was significantly shortened by about 70% after enzyme treatment [19], and in this case, CVs approaching 30 m/s could theoretically be resolved with the available nerve length (~1.5 cm after accounting for enzymatic shrinkage). For successful nerve stimulation, the stimulus intensity was set at 120% of activation, with the specific threshold varying by fiber type. Frequency following capacity was also tested using repeated pulse stimulation without changing the stimulus magnitude.

### 2.8 Recording Solutions

For current clamp recordings at room temperatures (21°C–23°C) the extracellular/bath solution consisted of (mM): 137 NaCl, 5.4 KCl, 1.0 MgCl_2_, 2.0 CaCl_2_, 10 glucose, 10 N‐2‐hydroxyethylpiperazine‐N′‐2‐ethanesulfonic acid (HEPES). The pH was adjusted to 7.3 with 1 N NaOH. The intracellular/pipette solution was prepared (mM): 6.0 NaCl, 50 KCl, 50 K_2_SO_4_, 5.0 MgCl_2_, and 10 HEPES. The pH was adjusted to 7.2 with 1 N KOH. Just prior to recording, Na‐GTP and Na‐ATP were added to the pipette solution.

### 2.9 Action Potential Simulation by Current Injection From Dynamic‐Current Clamp (DCC)

There is a technical limitation to assess the exact role of TTX‐S and TTX‐R Na^+^ channels in the development of AP depolarization and the sequential order of when they are kicked in during the depolarization process. Therefore, the dynamic‐current clamp (DCC) was employed to simulate AP trajectory by current injection where gNa0 and gNa1 represent the maximal conductance (in nS) of TTX‐S (mostly Nav1.7) and TTX‐R (mostly Nav1.8), respectively. To simplify the evaluation, identified myelinated A‐type NG neurons were used in this study as model neurons with TTX‐S Na^+^ expression only that could be easily blocked by 200 nM TTX (Figure S4).

Both A‐type and C‐type APs and their repetitive discharges could be simulated by adjusting the DCC input. Specifically, A‐type AP generation required only gNa0 input (representing TTX‐S/Nav1.7), whereas C‐type AP generation required combined gNa0 + gNa1 inputs (representing TTX‐S/Nav1.7 + TTX‐R/Nav1.8). The voltage‐dependent profiles of activation and inactivation for both channel subtypes were established by systematically adjusting these digitized inputs to closely approximate real recorded AP trajectories [11, 13, 15] (Figure S5). This iterative matching process ensured that the simulated APs reproduced key characteristics including depolarization rate, peak amplitude, and repolarization phase.

For determining the TTX‐S/TTX‐R channel density ratio, different approaches were used for A‐type and C‐type neurons. In A‐type neurons, current density calculation is straightforward as TTX‐S is the exclusive Na^+^ channel type, allowing direct calculation from whole‐cell capacitance (WCC) and maximal TTX‐sensitive current. For C‐type neurons, total WCC and maximal whole‐cell current were first determined, followed by isolation of the TTX‐R component through application of 200 nM TTX. The TTX‐S/TTX‐R ratio was then estimated through model simulation using gNa0 and gNa1 values that successfully reproduced the C‐type AP trajectory including both the depolarization phase and peak amplitude. This approach allows quantification of the relative functional expression of both channel subtypes.

### 2.10 Determination of TTX‐R Kick‐In Voltage

Displacement current phase plots were generated from two typical C‐type neurons from sliced preparations. This approach was chosen because displacement current analysis can reveal subtle changes in ionic conductances during AP development that may not be apparent in the voltage trajectory alone. The kick‐in voltage for the TTX‐R component was determined as follows: first, a tangent line (line #1) (Figure S6) was placed alongside the downward portion with visible inflection of the displacement current phase plot; second, a perpendicular line (line #2) was placed at the most obvious inflection point and intersected at the tangent line; third, a vertical line (line #3) was drawn through the intersection between line #1 and line #2, and the kick‐in voltage for the TTX‐R component was determined from the horizontal coordinate. This geometric method provides an objective, reproducible approach to identify the voltage at which TTX‐R channels begin contributing substantially to the total Na^+^ current. The inflection point in the displacement current phase plot represents the transition where additional conductance from TTX‐R channels is recruited during depolarization. The validity of this approach was confirmed by our DCC simulation experiments (see Section 3.4 and Figure 4) and pharmacological blockade studies (see Section 3.1) showing sequential activation of TTX‐S followed by TTX‐R channels.

### 2.11 Data Acquisition and Statistical Analysis

Statistical analysis and figure generation were performed using Microsoft Excel 2007 and Origin 7.0 (OriginLab, Northampton, MA, USA). All electrophysiological data generated by patch‐clamp recordings were analyzed with Clampfit (v10.3; Molecular Devices, Sunnyvale, CA, USA). Statistical analyses were only performed on studies that had at least 3 complete observations (*n*), as indicated in the figure legends. Both paired and unpaired Student's *t*‐tests were employed to assess the statistical significance. When necessary, one‐way ANOVA with post hoc Tukey test was utilized for comparisons among multiple data sets. Data are presented as mean ± SD. A *p*‐value < 0.05 was considered statistically significant.

## 3 Results

### 3.1 Relationship and Role of TTX‐S and TTX‐R Na^+^ Channel Currents in the Development of AP Depolarization and Repetitive Discharge in Unmyelinated C‐Type Neurons From Isolated and Slice Preparations

**FIGURE 1:**
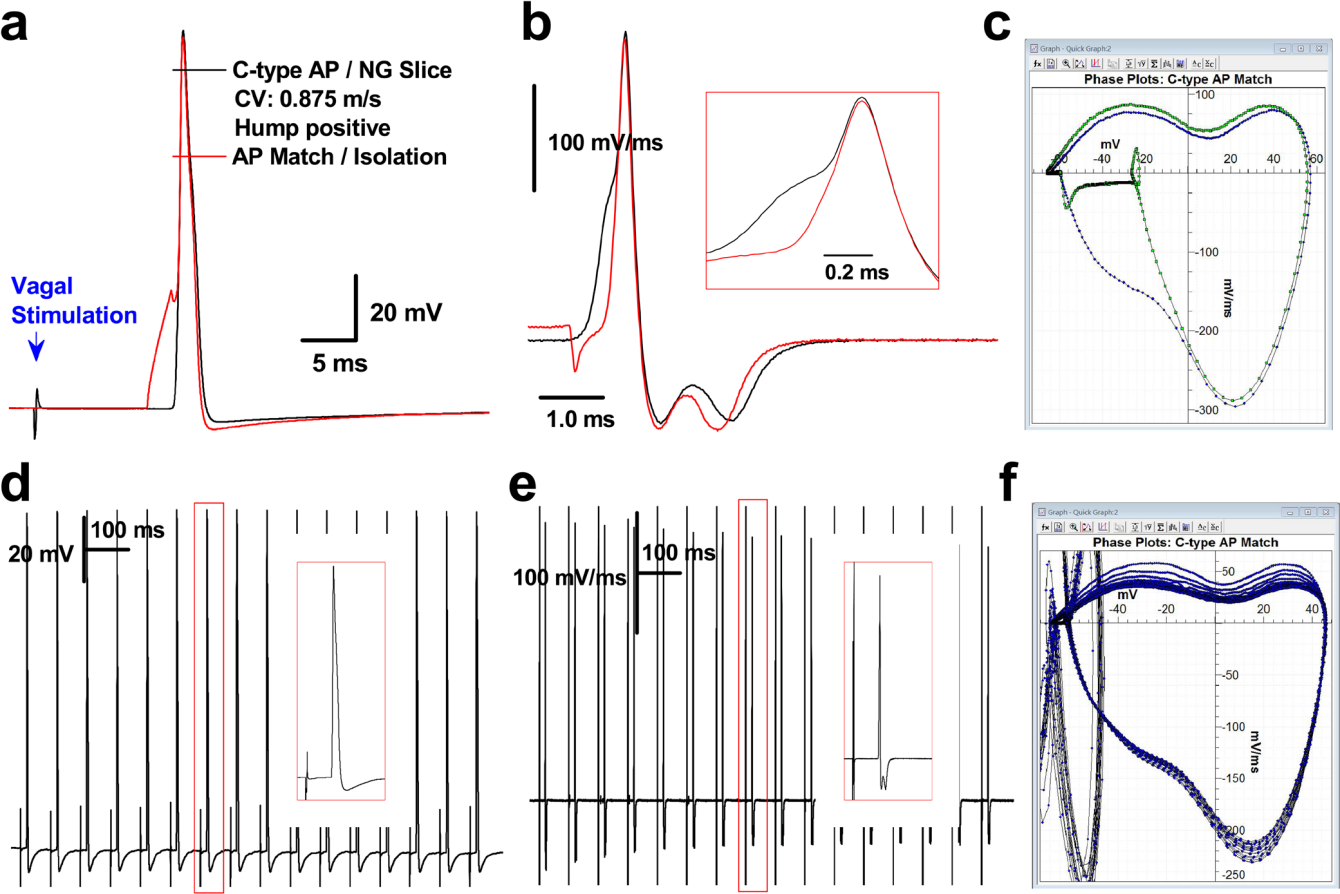
Phase plots combined with voltage derivatives revealed sequential kick‐in of TTX‐S and TTX‐R Na^+^ channel currents during depolarization. All APs were recorded from the same identified unmyelinated C‐type neuron evoked by brief pulse or vagal stimulation. (a) Action potentials (APs) from isolated and slice preparations; (b) voltage derivatives (dV/dt) plotted from APs illustrated in (a) and details of depolarization; (c) phase plots created from those APs illustrated in (a) (black: Sliced preparation; green: Isolated preparation); (d) discharge capability by repeated vagal stimulation; (e) voltage derivatives plotted from discharge shown in (d); (f) phase plots generated from discharges shown in (d).

It is well known that both TTX‐S and TTX‐R Na^+^ channel currents participate in the AP depolarization process [4, 20]; however, it is difficult to identify the role of each component, their sequential involvement, and the exact kick‐in time/voltage of TTX‐R following TTX‐S, which could previously only be inferred from voltage‐dependent activation profiles showing that TTX‐S activates sooner than TTX‐R. For this regard, we employed the displacement current phase plots (SF. 6), that is, the membrane current was plotted as the function of membrane voltage over the time course of AP conjugated with voltage derivatives generated directly from AP trajectory. Unmyelinated C‐type neurons represent the major population (70%–80% of total) in the nodose ganglia (NG) of adult rats, with proportions varying by sex [21]. These neurons functionally express both TTX‐S and TTX‐R Na^+^ currents, making them ideal for testing our hypothesis: that TTX‐S and TTX‐R Na^+^ channel subtypes are sequentially activated during AP depolarization, with TTX‐S initiating the depolarization and TTX‐ R being recruited at more depolarized voltages to support complete AP generation and maintain neuronal excitability. Both APs from isolated and sliced preparations exhibited brief, smooth upstrokes without visible indication of dual Na^+^ component involvement in the voltage trajectories (Figure 1a). Even with highly expanded time scales (Figure S7), the AP waveforms did not reveal sequential activation of TTX‐S and TTX‐R channels. However, voltage derivative analysis (Figure 1b) clearly revealed a biphasic upstroke pattern, particularly in APs evoked by vagal stimulation in sliced preparations (black trace) compared to isolated preparations (red trace). This dual‐component signature was even more evident in displacement current phase plots (Figure 1c, Figure S8), which displayed a distinct inflection point during the depolarization phase. The inflection point represents the transition where TTX‐R channels begin contributing to the total Na^+^ current following initial TTX‐S activation. These findings demonstrate that both TTX‐S and TTX‐R components contribute sequentially to AP depolarization, enabling quantification of TTX‐R kick‐in voltage following TTX‐S activation. Additionally, discharge capability elicited by repeated vagal stimulations was also tested and repetitively fired APs, displacement phase plots and derivatives generated from those APs trajectories fully supported our observations (Figure 1d–f).

### 3.2 Kick‐In Voltage of TTX‐R Following TTX‐S Na^+^ Component Negatively Correlated With Functional Expression of Na^+^ Channels

**FIGURE 2:**
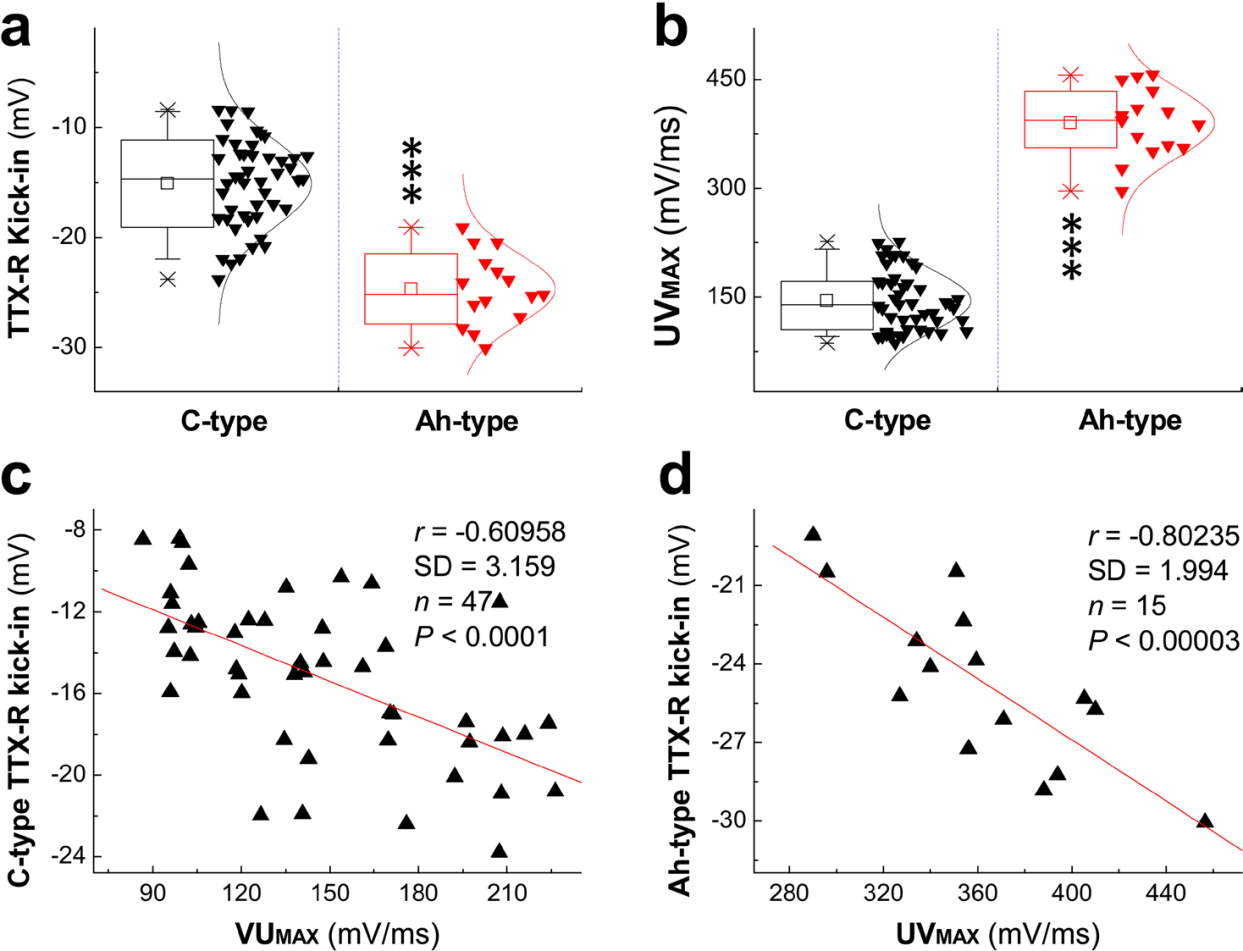
The sequential kick‐in time of TTX‐R following TTX‐S Na^+^ channel current and correlation between kick‐in voltage and maximal up‐stroke velocity (UVMAX) in myelinated Ah‐type and unmyelinated C‐type neurons. (a) Kick‐in voltage for both afferent fiber types; (b) UVMAX for both afferent fiber types; (c) correlation for C‐types; (d) correlation for Ah‐types. Data are expressed as mean ± SD, *n* = 47 for C‐type and 15 for Ah‐type; ***p < 0.001 vs. C‐type.

Averaged data showed that the kick‐in voltage (negative value) in Ah‐type neurons was significantly more negative than in C‐type neurons (Figure 2a), whereas the maximal up‐stroke velocity (UV_MAX_, Figure 2b), which indirectly represents the functional expression of Na^+^ channels and correlates with fast conduction velocity, was higher in Ah‐type compared to C‐type neurons [18, 22]. This observation suggests that the variation in kick‐in voltage is likely attributable to differences in functional Na^+^ channel expression, which can be quantified by UV_MAX_ given its close relationship to the rate of depolarization. Correlation analysis confirmed a significant negative correlation between kick‐in voltage and UV_MAX_ in both C‐type and Ah‐type neurons (Figure 2c,d).

The aforementioned findings suggest that TTX‐R components in both C‐type and Ah‐type neurons may activate earlier than observed, as overlapping activation of TTX‐S and TTX‐R components—particularly in neurons with faster depolarization—could mask the true TTX‐R kick‐in voltage. To gain quantitative insights into this phenomenon, we screened a large number of neurons from sliced preparations and identified two representative examples (Figure S9) that met all C‐type classification criteria (CVs < 1.0 m/s at room temperature and characteristic AP waveform features) but exhibited unusually small TTX‐S components. In these neurons with minimal TTX‐S expression (Figure S9), the first Na^+^ component amplitude in voltage derivatives was dramatically smaller (approximately 20 and 40 mV/ms) compared to C‐type neurons with typical TTX‐S expression levels in the main dataset (≥ 60 mV/ms; Figure 2, Figure S8). Correspondingly, the TTX‐R kick‐in voltages in these cases with minimal TTX‐S were more negative (approximately −39.9 and −33.8 mV in Figure S9) compared to neurons with robust TTX‐S expression (−9.65 and −19.1 mV in Figure S8).

These observations, confirmed by displacement current phase plot analysis, demonstrate an inverse relationship between TTX‐S component amplitude and TTX‐R kick‐in voltage, indicating that the two channel subtypes work cooperatively through compensatory mechanisms. This compensatory relationship is further supported by the strong correlation between UV_MAX_ and kick‐in voltage (Figure 2c,d). Intriguingly, we recorded a very rare C‐type neuron from sliced preparation (Figure S10) in which the first (TTX‐S) component was only approximately 5 mV/ms in the voltage derivative, with a corresponding kick‐in voltage of approximately −40 mV. This extreme case suggests that when TTX‐S expression is minimal, the measured kick‐in voltage may approach the true activation threshold for TTX‐R channels, implying that typical C‐type neurons with robust TTX‐S expression may mask earlier TTX‐R recruitment due to current overlap.

### 3.3 Sequential Activation of TTX‐S and TTX‐R Currents in Myelinated Ah‐Type Neurons

**FIGURE 3:**
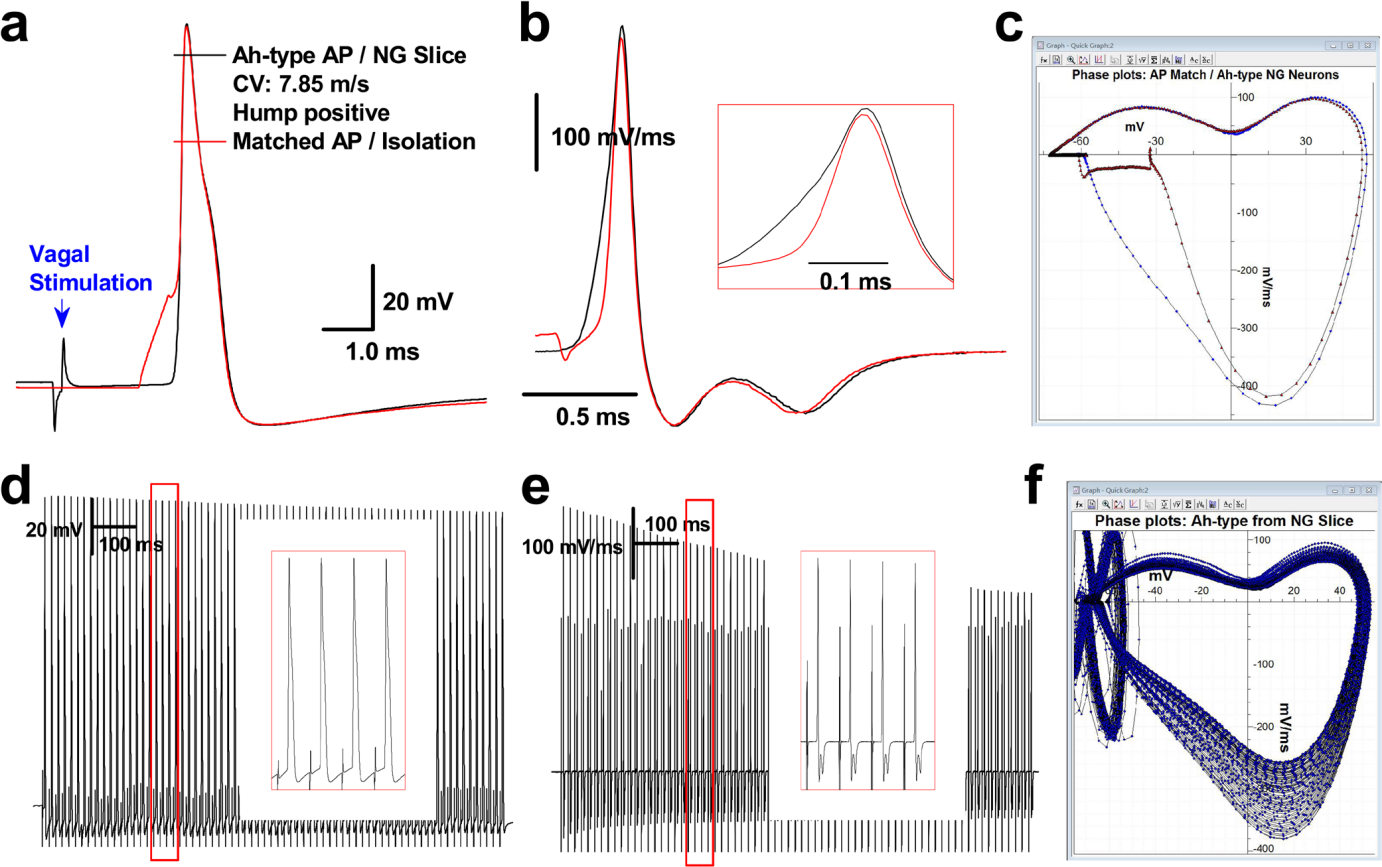
Phase plots combined with voltage derivatives revealed sequential kick‐in of TTX‐S and TTX‐R Na^+^ channel currents during depolarization. All APs were recorded from the same identified myelinated Ah‐type neuron evoked by brief pulse or vagal stimulation. (a) Action potentials (APs) from isolated and slice preparations; (b) voltage derivatives plotted from APs illustrated in (a) and details of depolarization; (c) phase plots created from those APs illustrated in (a); (d) discharge capability by repeated vagal stimulation; (e) voltage derivatives plotted from discharge shown in (d); (f) phase plots generated from discharges shown in (d).

Even though myelinated Ah‐type neurons have specifically been observed in adult female rats [18] with nearly equal population with A‐types ranging from 10% to 20%, these neurons are key players in sexual dimorphism of neurocontrol of blood pressure [23, 24, 25, 26] and baroreflex afferent function [21, 27], cardiac pain [28, 29], and asthma [30, 31]. Interestingly, these Ah‐type neurons functionally express Nav1.7, Nav1.8, and Nav1.9 [22] and display a similar waveform trajectory to C‐types, particularly in the repolarization; thus, Ah‐types also bring our attention to whether or not TTX‐S and TTX‐R play a similar role in the AP depolarization. For this regard, a similar set of experiments was conducted in Ah‐types identified by the waveform characters for isolated and the afferent fiber CVs for sliced preparations. Consistent data were observed in a group of Ah‐type neurons (Figure 3a–f). Compared to C‐type neurons, the inflection point indicating TTX‐R kick‐in following the TTX‐S Na^+^ component was less prominent in Ah‐type neurons, even in sliced preparations (black trace, Figure 3b inset). This is attributable to the faster UVMAX and stronger correlation between Na^+^ components in Ah‐type neurons, which presents a challenge for accurately quantifying the TTX‐R kick‐in voltage. Nonetheless, the kick‐in voltage can be determined by expanding the time or voltage scale in the voltage derivative or displacement current phase plot.

### 3.4 Simulation of AP Depolarization by Current Injection of TTX‐S/gNa0 and TTX‐R/gNa1 Na^+^ Components Using Dynamic‐Current Clamp

**FIGURE 4:**
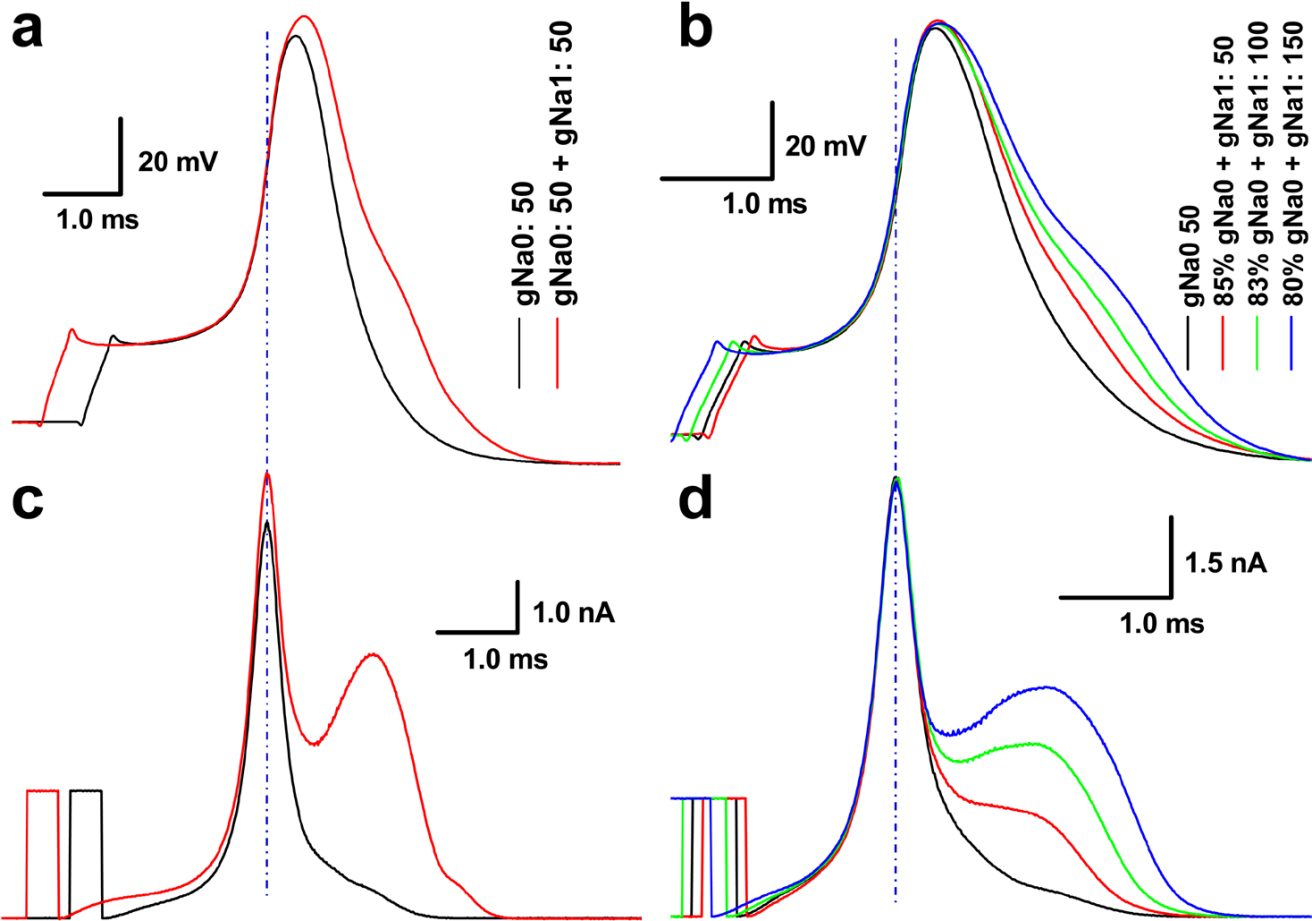
Action potential (AP) simulation by dynamic current‐clamp (DCC) reveals coordinated roles of TTX‐S and TTX‐R Na^+^ channel currents. Dynamic current‐clamp (DCC) simulation demonstrating the relationship between TTX‐S (gNa0) and TTX‐R (gNa1) Na^+^ channel currents during AP depolarization. gNa0 and gNa1 represent digitized conductance inputs from DCC, where the numerical values (e.g., gNa0: 50 nS) indicate the simulated conductance amplitude. (a) APs generated by DCC current injection with gNa0 alone (black, simulating A‐type AP) and combined gNa0 + gNa1 (red, simulating C‐type AP); (b) series of C‐type APs generated by progressively increasing gNa1 while proportionally decreasing gNa0 to maintain similar peak amplitudes. This demonstrates that TTX‐S and TTX‐R components work in a coordinated, compensatory manner to regulate AP peak. (c) Corresponding DCC current injection profiles from panel (a). (d) Corresponding DCC current injection profiles from panel (b). All traces are temporally aligned by horizontal shifting for easier comparison. Vertical blue dashed lines indicate the voltage at which TTX‐R channel are recruited following initial TTX‐S activation.

In order to learn how TTX‐S and TTX‐R Na^+^ components coordinately fine‐tune the depolarization in visceral sensory neurons co‐expressing both currents, we introduced dynamic current‐clamp (DCC) here [11, 13] attempting to simulate the role of each Na^+^ component or combination in the development of AP depolarization. Myelinated A‐type neurons from isolated preparations were chosen as a model system because these neurons express exclusively TTX‐S (mostly Nav 1.7) channels, which can be completely blocked by 200 nM TTX (Figure S4). After TTX blockade, both A‐type and C‐type APs could be simulated by DCC current injection, where gNa0 and gNa1 represent TTX‐S and TTX‐R components, respectively (Figure S4). Due to the amount of TTX‐S functional expression represented by the 1st component of the voltage derivative varying upon the tested A‐type neurons (Figures S8–S10), the range of injected gNa0 was between 20 and 120 nS from DCC, the average was about 50 (Figure S4). Interestingly, after the A‐type AP was matched (Figure 4a, black trace) by gNa0 50, the gNa1 50 injection on top of gNa0 caused a significant increase in the peak of AP (Figure 4a, red trace); however, by looking deeply into the corresponding DCC current injection profiles (Figure 4c), the 1st peak representing gNa0 was also increased correspondingly after adding gNa1 (Figure 4c), suggesting that an initiated part of AP depolarization is contributed to by gNa0 and the later portion of AP depolarization is jointly contributed to by gNa0 and gNa1 at the point shown by the vertical dash‐dot line (Figure 4a,c). Importantly, this speculation was further supported by observations during the simulation of C‐type APs with varying TTX‐R contributions. As gNa1 was progressively increased to generate C‐type APs with larger repolarization humps—a characteristic feature resulting from TTX‐R reactivation during repolarization (Figure 4b)—gNa0 had to be correspondingly decreased to maintain similar peak amplitudes in both DCC current injection profiles and AP waveforms (Figure 4b,d). This compensatory adjustment demonstrates the coordinated relationship between TTX‐S and TTX‐R components in determining AP characteristics. Taken together, these data indicate that both TTX‐S and TTX‐R (mostly Nav1.7 and Nav1.8) Na^+^ components contribute to AP depolarization in a coordinated (*r* = 0.909, *n* = 5; *p* < 0.0001) and compensatory manner.

### 3.5 Simulation of Firing Capability by Current Injection of TTX‐S/gNa0 and TTX‐R/gNa1 Na^+^ Components Using Dynamic‐Current Clamp (DCC)

The frequency of repetitive discharge of visceral sensory neurons is a direct parameter to evaluate their neuronal excitability, and the sensory information is encoded into trains of repetitively firing APs. Our previous data have shown that A‐type and C‐type neurons fire at approximately 150 and 50 Hz, respectively, in sliced preparation [32, 33], and at even lower frequencies in isolated preparations [31, 34]. This reduced firing capability in C‐type neurons suggests that TTX‐R Na^+^ channels may constrain repetitive firing—a hypothesis difficult to test directly with conventional patch‐clamp recording.

To investigate how TTX‐R recruitment dynamics affect firing capability, we used DCC to simulate C‐type APs (as in Figure 4b) with combined gNa0/gNa1 injection, then tested repetitive discharge evoked by step depolarization (Figure S2). To maintain consistent AP peak amplitude across trials, gNa0 and gNa1 amplitudes were held constant while only the integrated step size was varied (Figure S11), allowing us to isolate the effects of TTX‐R recruitment dynamics on firing capability. The integrated step size parameter controls the temporal dynamics of gNa1 activation in the DCC model—larger step sizes delay and broaden TTX‐R current recruitment (SF. 11), effectively simulating delayed channel activation. This approach allowed us to systematically examine whether delayed TTX‐R recruitment impairs repetitive firing capability.

**FIGURE 5:**
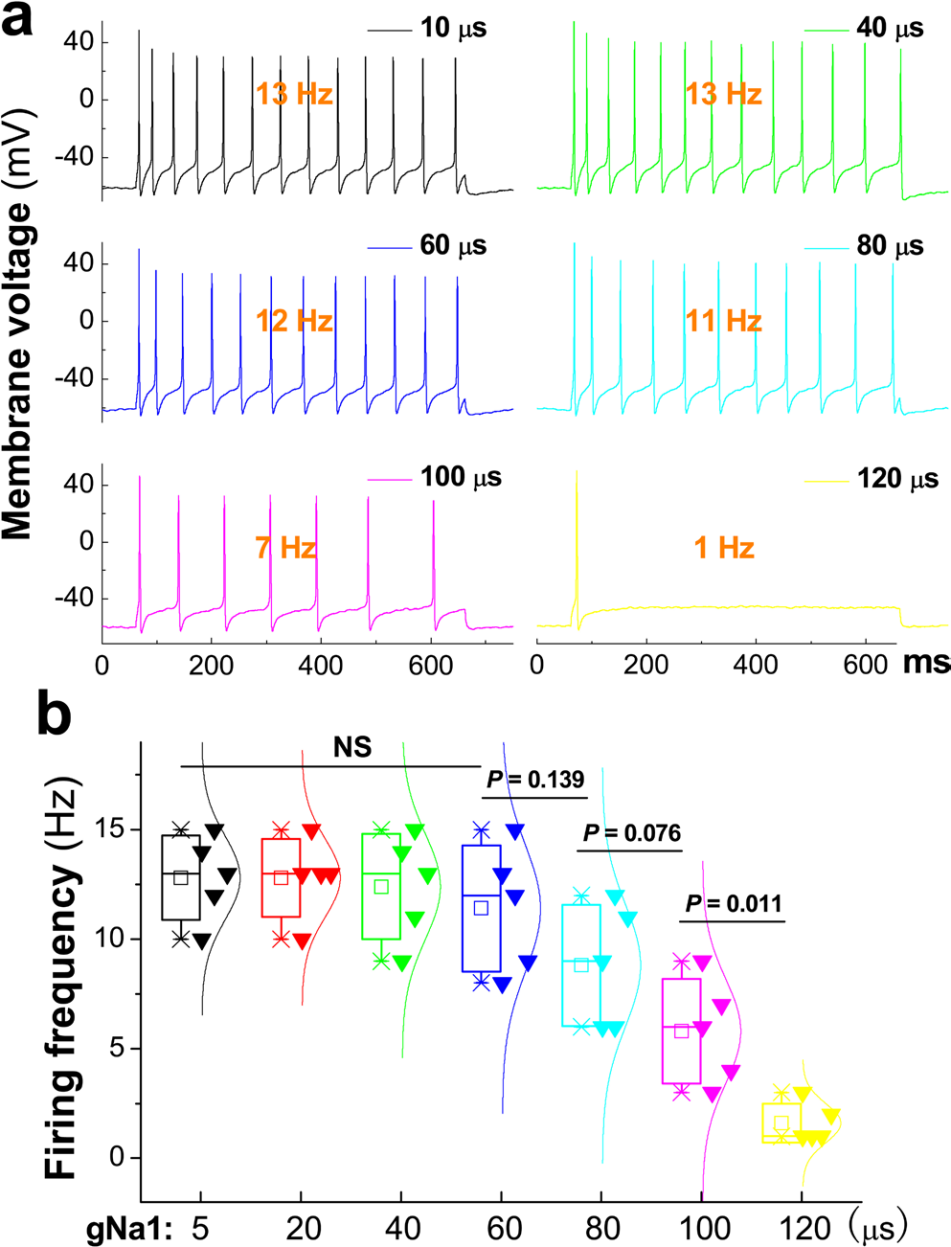
TTX‐R Na^+^ component limits repetitive discharge capability with increasing step size. (a) Gradual reduction of firing frequency during repetitive discharge with increasing integrated step size (μs) for gNa1 superimposed on basal gNa0 via DCC current injection; (b) summary of relationship between firing frequency and gNa1 step size. Data are expressed as mean ± SD, *n* = 5 recordings. NS, no significance.

**FIGURE 6:**
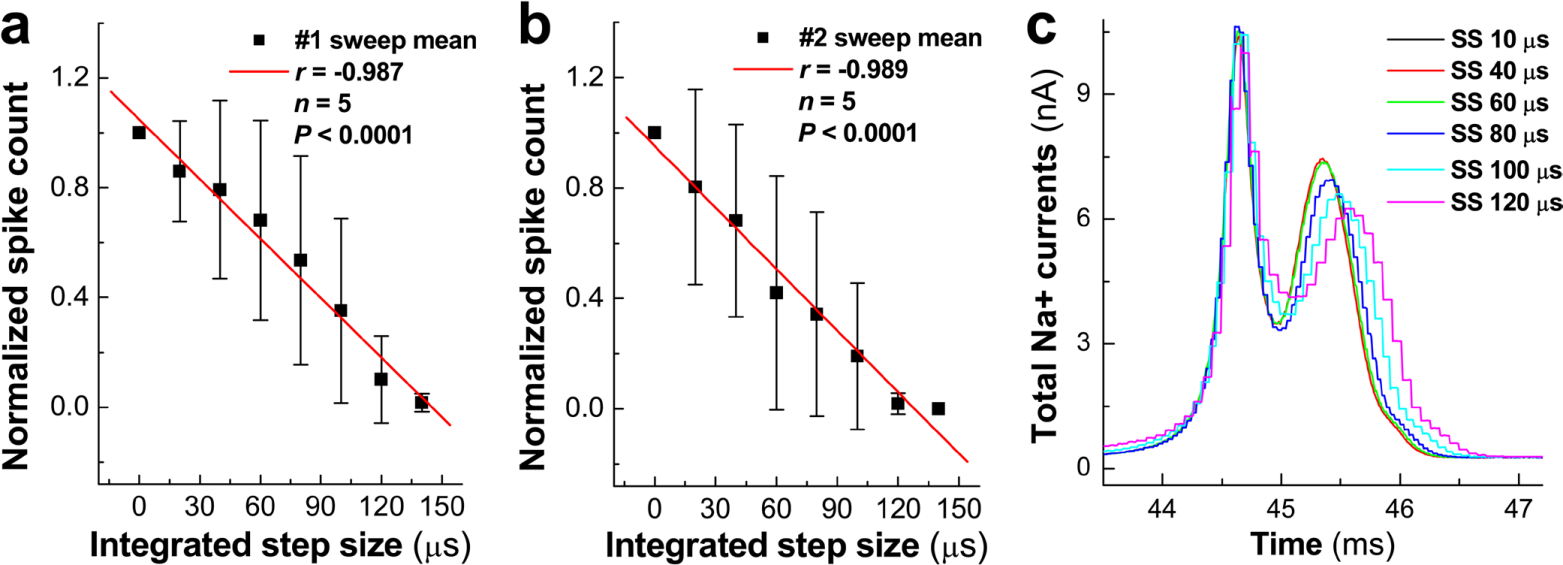
Correlation between normalized spike number and integrated step size (SS) and DCC current injection profiles. (a, b) Correlations between normalized spike number and SS for both sweeps; (c) representative DCC current injection profiles corresponding to recordings shown in Figure 5a, showing the first peak (gNa0/TTX‐S) and second peak (gNa1/TTX‐R) of total Na^+^ current. Data are expressed as mean ± SD, *n* = 5 recordings.

Interestingly, the frequency of repetitive discharges was reduced in a step size‐dependent manner (Figure 5a,b) without changing gNa0 and gNa1 amplitudes, and strong negative correlations were confirmed between spike number and integrated step size (Figure 6a,b) in both sweeps (*r* = −0.987 and −0.989, *n* = 5, *p* < 0.0001).

Surprisingly, analysis of the total Na^+^ currents during individual APs revealed distinct effects on TTX‐S versus TTX‐R components. The first current peak (Figure 6c), representing gNa0/TTX‐S contribution, remained unchanged across different step sizes (*p* > 0.05, both paired *t*‐test and ANOVA). In contrast, the second current peak, representing gNa1/TTX‐R contribution, was dramatically reduced in a step size‐dependent manner (Figure 6c, Figure S12). This reduction was particularly evident at step sizes ≥ 80 μs, which corresponds to the step sizes (80–120 μs) showing impaired repetitive firing in the recordings (Figure 5a) and reduced spike numbers in the analysis (Figure 5b). Representative current traces showing this step size‐dependent effect on both peaks are illustrated in SF. 12. Additionally, both peaks were shifted to the right, with the 2nd one being shifted the most. These data indicate that the coordination of TTX‐S and TTX‐R is critical for maintaining neuronal excitability in C‐type neurons, and that increased step size delays TTX‐R recruitment (evidenced by rightward shift), leading to reduced TTX‐R current amplitude and disrupted channel coordination. From this perspective, TTX‐R (mostly Nav1.8) Na^+^ channels are fundamental for sensory reflex afferent function.

## 4 Discussion

### 4.1 Major Findings

Our combined experimental and modeling approach reveals that TTX‐S and TTX‐R Na^+^ channels play distinct but coordinated roles in nodose sensory neuron excitability. The major findings are: (1) TTX‐sensitive (TTX‐S) Na^+^ channels initiate AP depolarization, whereas TTX‐R channels are recruited at more depolarized voltages, with kick‐in voltage inversely correlated with TTX‐S expression level; (2) TTX‐R channels are reactivated during repolarization, generating the characteristic C‐type hump that can be reproduced by sequential gNa1 injection; (3) AP peak amplitude is maintained through compensatory adjustment between gNa0 and gNa1 inputs; and (4) TTX‐R channels sustain neuronal excitability during repetitive firing, as firing capability declines when TTX‐R recruitment is delayed by increased integrated step size.

### 4.2 Advantages

Several advantages of current investigation include: (1) A perfect cell model, that is, AP could be elicited from A‐type NG that expresses TTX‐S only, so injected gNa0 or gNa1 could be accurately controlled by DCC; (2) A‐type APs could be elicited from both isolated and sliced preparations. APs from sliced preparation more closely reflect physiological conditions, as they are evoked by vagal stimulation and depolarized directly from the resting membrane potential. Voltage derivatives and displacement current phase plots generated from the same APs of both preparations provide detailed insights into TTX‐S and TTX‐R dynamics during depolarization; and (3) injected gNa0/TTX‐S or gNa1/TTX‐R or in combination from dynamic current‐clamp (DCC) could be quantified to figure out the functional expression of both Na^+^ channels, coordination and dynamic changes during depolarization in tested NG neurons.

### 4.3 Limitations and Future Perspectives

Electrophysiological recordings were conducted at room temperature, which affects absolute kinetic values but preserves the relative timing relationships between TTX‐S and TTX‐R channel subtypes. It is therefore critical to include a basal gNa0 injection when simulating APs, as the TTX‐S component is indispensable for faithful AP reproduction. Although an AP can be elicited using gNa1 alone in C‐type neurons, the resulting waveform parameters fall outside normal physiological ranges—the depolarization rate is substantially slower, the required stimulus intensity is excessive, and the overall waveform becomes distorted. This confirms that TTX‐S is essential for maintaining normal AP characteristics, and that the kick‐in voltage for the TTX‐R component, whereas closely approximated, remains an estimate in our DCC simulations. Considering those advantages mentioned above, these observations will add tremendous solid information in the field of neurosciences to fully understand visceral afferent reflex function under physiological and disease conditions and will benefit pharmacological interventions.

## 5 Conclusion

Taken all these data together, our current observations have demonstrated for the first time in our best acknowledgment that, by combined use of patch‐clamp and DCC modeling, both TTX‐S and TTX‐R sequentially and dynamically participate in the AP depolarization by fine tuning gNa0/TTX‐S and/or gNa1/TTX‐R injection in a tight coordination manner to retain the peak of AP and the neuroexcitability. These works definitely provide very insightful information to the field of neuroscience for further understanding the role of TTX‐S and TTX‐R Na^+^ channels in visceral sensory aspects from a special angle of real recordings and model simulations.

## Author Contributions

**Wu Di:** conceptualization, investigation, writing – review and editing, project administration, funding acquisition. **Dou Tian‐min:** data curation, writing – review and editing. **Zhang Jing‐ran:** conceptualization, methodology, writing – original draft, investigation. **Zhang Hong‐fei:** methodology. **Li Xing‐yu:** investigation, writing – review and editing. **Li Bai‐yan:** investigation, writing – review and editing, project administration. **Fu Hui‐xiao:** methodology, conceptualization, investigation, writing – review and editing.

## Conflicts of Interest

The authors declare no conflicts of interest.

## Supporting information


**Figure S1:** Brief constant‐pulse current protocol used to elicit single action potentials (APs), with representative parameters of ~500 μs duration and ~400 pA amplitude. Multiple sequential sweeps were applied with an inter‐pulse interval of 10–30 s to allow complete recovery from refractoriness. Vertical axis: output current (pA); horizontal axis: time (ms).
**Figure S2:** Step‐current depolarization protocol used to evoke repetitive discharge, with step duration of 500–1000 ms, initial amplitude ~200 pA, and incremental increases in subsequent steps; inter‐step interval 10–30 s. Vertical axis: current (pA); horizontal axis: time (ms).
**Figure S3:** Monophasic constant‐current pulse protocol applied to the vagus nerve via a bipolar electrode in sliced preparations. Pulse duration was individually adjusted for each preparation to account for variations in nerve length and surrounding tissue. Vertical axis: stimulus voltage (V); horizontal axis: time (ms).
**Figure S4:** Myelinated A‐type isolated neurons as a model for dynamic current‐clamp (DCC) simulation. (A) Representative APs before (black) and after (red) 200 nM tetrodotoxin (TTX), together with the DCC‐simulated AP generated by gNa0 injection (green). (B) Voltage derivatives generated from the APs in (A). (C) Enlarged *Y*‐axis scaling of (B). Complete TTX blockade confirms exclusive TTX‐S expression in A‐type neurons, and the close match between control and DCC‐simulated derivatives validates the DCC modeling approach. The horizontal time scale (1.0 ms) in (A) applies to all panels.
**Figure S5:** Voltage‐dependent activation and inactivation profiles of gNa0 (TTX‐S, mostly Nav1.7) and gNa1 (TTX‐R, mostly Nav1.8) conductances established through DCC simulation. The rightward shift of gNa1 activation relative to gNa0 indicates that TTX‐R activates at more depolarized potentials. Conductance was normalized to 0–1. Curves: black, gNa0 activation; red, gNa0 inactivation; green, gNa1 activation; blue, gNa1 inactivation.
**Figure S6:** Geometric method for determining the TTX‐R kick‐in voltage from displacement current phase plots, illustrated in two representative C‐type neurons from sliced preparations: (1) tangent line (line #1) drawn along the downward phase with visible inflection; (2) perpendicular line (line #2) drawn at the most prominent inflection point, intersecting line #1 at point α; and (3) vertical line (line #3) drawn through point α, with the intersection with the horizontal axis defining the kick‐in voltage. *X*‐axis: membrane potential (mV); *Y*‐axis: voltage derivative (mV/ms).
**Figure S7:** Time‐expanded AP upstrokes from sliced preparations. Left: C‐type neuron. Middle: Ah‐type neuron. Right: overlay (blue, C‐type; red triangles, Ah‐type). Even with expanded temporal resolution, the upstrokes appear smooth without a visible dual TTX‐S/TTX‐R signature, demonstrating the necessity of voltage‐derivative and displacement current phase plot analyses.
**Figure S8:** Two representative C‐type neurons from sliced preparations with typical TTX‐S expression. Left panels: AP trajectories (red) and voltage derivatives (black). Right panels: displacement current phase plots. Horizontal dashed lines indicate the voltage‐derivative amplitude at the inflection, and vertical dashed lines indicate the TTX‐R kick‐in voltage.
**Figure S9:** Two representative C‐type neurons with unusually small TTX‐S components. The first Na^+^ component in the voltage derivatives was markedly smaller than in typical recordings, with correspondingly more negative TTX‐R kick‐in voltages (approximately −39.9 and −33.8 mV). These neurons were excluded from the main kick‐in voltage analysis. Panel arrangement and annotations are the same as in Figure S8.
**Figure S10:** A rare C‐type neuron from sliced preparation that met all classification criteria (CV < 1.0 m/s; characteristic waveform) but showed an exceptionally small first Na^+^ component (~5 mV/ms). Left: AP trajectory (red) and voltage derivative (black) showing that TTX‐R was recruited just before the first component returned to baseline. Right: displacement current phase plot with a kick‐in voltage of approximately −40 mV, substantially more negative than in typical C‐type neurons. This extreme case suggests that when TTX‐S expression is minimal the measured kick‐in voltage may approach the true activation threshold of TTX‐R channels. Excluded from main dataset analysis.
**Figure S11:** Representative gNa1 current injections from DCC with different integrated step sizes (left to right: 10, 60, and 100 μs). Total gNa1 amplitude is identical across conditions; only the integrated step size varies, so that increasing step size delays and broadens the current profile, affecting TTX‐R recruitment dynamics. Axes: time (ms) versus current (nA).
**Figure S12:** Representative total Na^+^ currents from DCC inputs with integrated step sizes ranging from 10 to 120 μs, showing the first peak (gNa0/TTX‐S) and the second peak (gNa1/TTX‐R). Color‐coded traces: black, 10 μs; red, 40 μs; green, 60 μs; blue, 80 μs; pink, 100 μs; orange, 120 μs. The first peak remained relatively stable, whereas the second peak progressively decreased and shifted rightward with increasing step size, supporting the analysis presented in Figure 6c. Axes: time (ms) versus total Na^+^ current (nA).

## Data Availability

The data that support the findings of this study are available from the corresponding author upon reasonable request.
