# Supplementary material for: TTX‐S/TTX‐R Na+ Currents Coordinately Fine‐Tune Depolarization/Firing Capability Revealed by Voltage Derivatives/Displacement Current Phase Plots With Dynamic Current‐Clamp Simulation in Rat Visceral Sensory Neurons
Source: Acta Physiol (Oxf). 2026 May 3;242:e70239. doi: 10.1111/apha.70239 (PMC13136411; doi:10.1111/apha.70239)
Supplement: Supplementary file 1 — Figure S1: Brief constant‐pulse current protocol used to elicit single action potentials (APs), with representative parameters of ~500 μs duration and ~400 pA amplitude. Multiple sequential sweeps were applied with an inter‐pulse interval of 10–30 s to allow complete recovery from refractoriness. Vertical axis: output current (pA); horizontal axis: time (ms). Figure S2: Step‐current depolarization protocol used to evoke repetitive discharge, with step duration of 500–1000 ms, initial amplitude ~200 pA, and incremental increases in subsequent steps; inter‐step interval 10–30 s. Vertical axis: current (pA); horizontal axis: time (ms). Figure S3: Monophasic constant‐current pulse protocol applied to the vagus nerve via a bipolar electrode in sliced preparations. Pulse duration was individually adjusted for each preparation to account for variations in nerve length and surrounding tissue. Vertical axis: stimulus voltage (V); horizontal axis: time (ms). Figure S4: Myelinated A‐type isolated neurons as a model for dynamic current‐clamp (DCC) simulation. (A) Representative APs before (black) and after (red) 200 nM tetrodotoxin (TTX), together with the DCC‐simulated AP generated by gNa0 injection (green). (B) Voltage derivatives generated from the APs in (A). (C) Enlarged Y‐axis scaling of (B). Complete TTX blockade confirms exclusive TTX‐S expression in A‐type neurons, and the close match between control and DCC‐simulated derivatives validates the DCC modeling approach. The horizontal time scale (1.0 ms) in (A) applies to all panels. Figure S5: Voltage‐dependent activation and inactivation profiles of gNa0 (TTX‐S, mostly Nav1.7) and gNa1 (TTX‐R, mostly Nav1.8) conductances established through DCC simulation. The rightward shift of gNa1 activation relative to gNa0 indicates that TTX‐R activates at more depolarized potentials. Conductance was normalized to 0–1. Curves: black, gNa0 activation; red, gNa0 inactivation; green, gNa1 activation; blue, gNa1 inactivation. Figure S6: [file APHA-242-e70239-s001.docx]

**Online only supplemental materials**

**TTX-S/TTX-R Na^+^**  **currents coordinately fine-tune depolarization/firing capability revealed by voltage derivatives/displacement current phase plots with dynamic current-clamp simulation in rat visceral sensory neurons**

Zhang Jing-ran^1, 2^*, Fu Hui-xiao^2,^ *, Li Xing-yu^2^, Zhang Hong-fei^2^, Dou Tian-min^2^, Li Bai-yan^2, 🖂^, Wu Di^1, 🖂^

**Supplemental figures (SFs) and legends** :

**Figure S1**

^
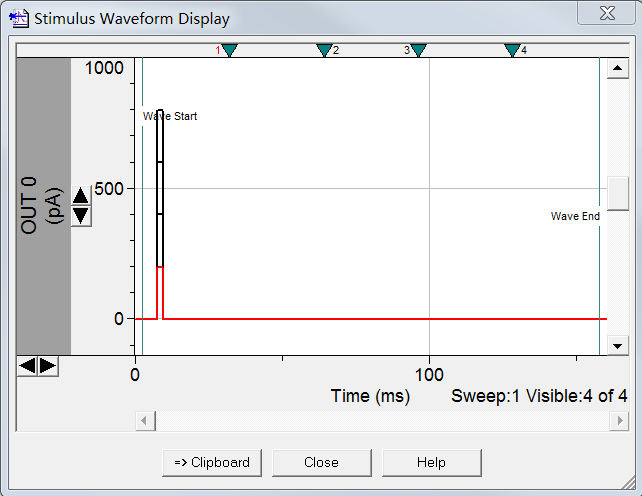
^

**Figure S1 Brief current pulse protocol for eliciting single action potentials.** Representative screenshot from the Stimulus Waveform Display showing the brief constant pulse protocol used to elicit single action potentials from isolated and sliced preparations. The waveform shows a brief current pulse with standard parameters: pulse duration ~500 μs (0.5 ms), amplitude ~400 pA. The display shows "Sweep 1 of 4," indicating multiple sequential stimulations with adjustable inter-pulse intervals (typically 10-30 s) to allow complete recovery from refractoriness. The vertical axis represents output current (OUT 0, in pA) and the horizontal axis represents time (in ms). This protocol corresponds to the "First" stimulus protocol described in Methods section 2.7.

**Figure S2**

^
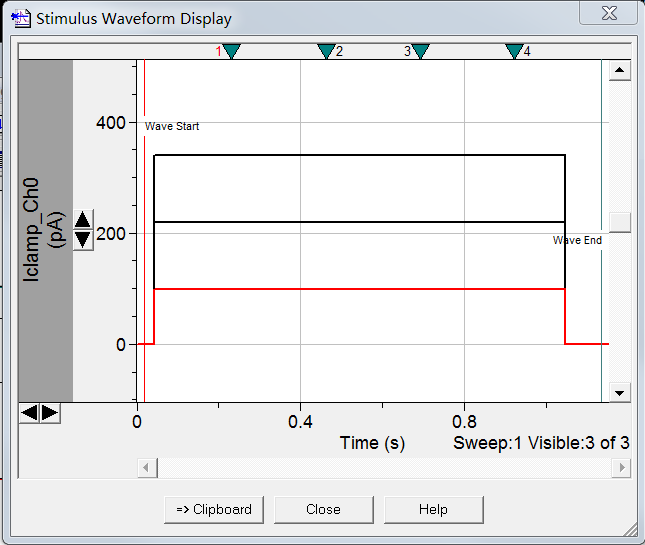
^

**Figure S2 Step current depolarization protocol for assessing repetitive discharge**. Representative screenshot from stimulation software showing the step current depolarization protocol used to evoke repetitive discharge in isolated preparations. Standard parameters: step duration 500-1000 ms, initial step amplitude ~200 pA, with incremental increases in subsequent steps. The inter-step interval was individually adjusted for each recording (typically 10-30 s). The display shows the configuration of step parameters including duration, amplitude, and increment size. Axes: vertical axis represents current amplitude (pA); horizontal axis represents time (ms). This protocol corresponds to the "Second" stimulus protocol described in Methods section 2.7.

**Figure S3**

^
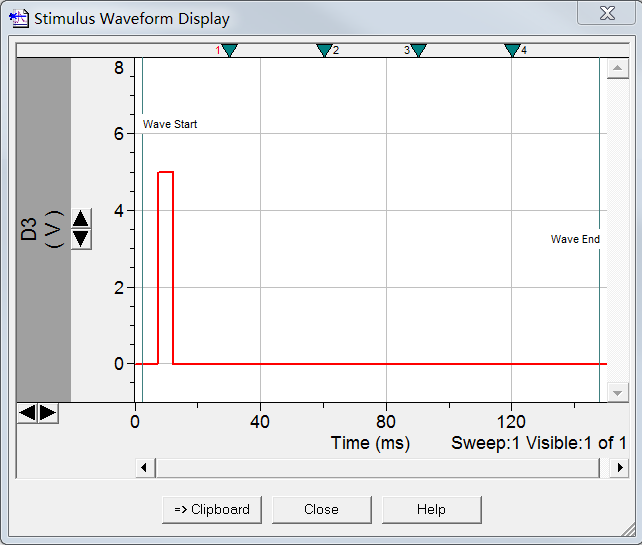
^

**Figure S3 Vagal nerve stimulation protocol with variable pulse duration**. Representative screenshot from stimulator interface showing the monophasic constant current pulse used for vagal nerve stimulation in sliced preparations. The waveform displays a single pulse (vertical axis: D3 in volts; horizontal axis: Time in ms). The pulse duration was individually adjusted for each slice preparation due to variations in nerve properties between animals. This protocol corresponds to the "Third" stimulus protocol described in Methods section 2.7.

**Figure S4**


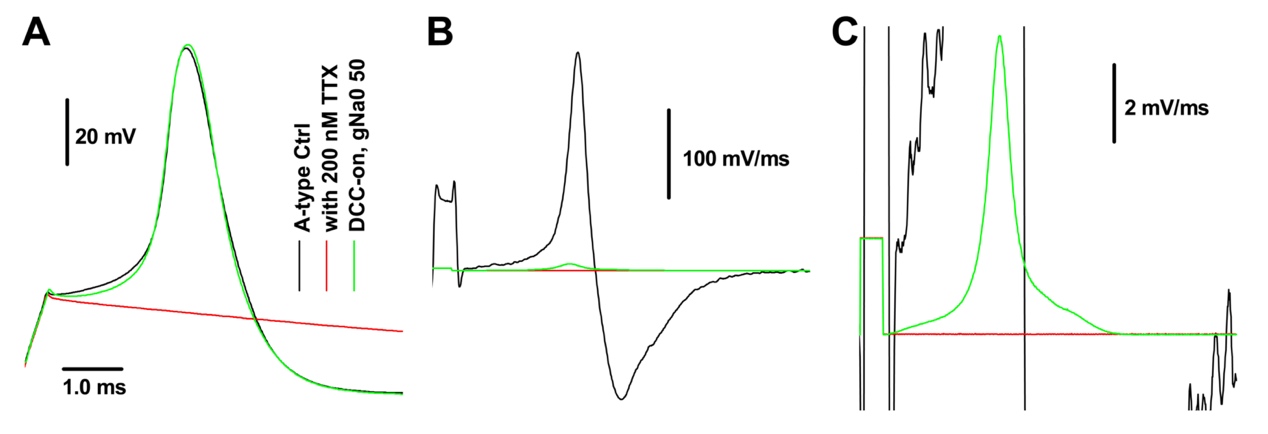


**Figure S4 Myelinated A-type isolated neurons as a model for dynamic current-clamp simulation**. **A**. Representative action potential (AP) recordings before (black) and after (red) 200 nM tetrodotoxin (TTX), and simulated AP by gNa0 current injection from dynamic current-clamp (DCC) (green). **B**. Voltage derivatives generated from APs shown in panel A: before TTX (black), with 200 nM TTX (red), and DCC-simulated AP with gNa0 injection (green). **C**. Enlarged Y-axis scaling of panel B to show detailed comparison. Complete TTX blockade (red traces in A-B) confirms exclusive TTX-sensitive (mostly Nav1.7) Na⁺ channel expression in A-type neurons. The close match between control (black) and DCC-simulated (green) derivatives validates the DCC modeling approach. Scale bars are indicated in each panel. The horizontal time scale in panel A (1.0 ms) applies to panels B and C. Note that although the DCC-simulated AP waveform closely matches the native AP (panel A), the voltage derivatives differ (panel B) because DCC injects current rather than directly controlling membrane voltage, resulting in different depolarization kinetics—a fundamental distinction between biological and computationally simulated APs.

**Figure S5**


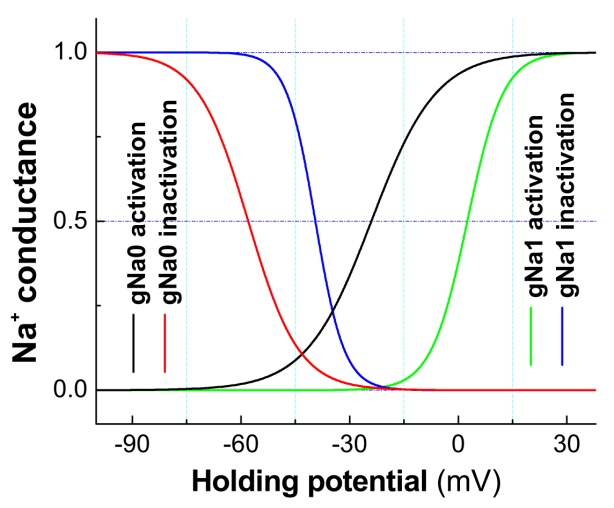


**Figure S5 Voltage-dependent activation and inactivation profiles of TTX-S and TTX-R Na^+^ currents**. Voltage-dependent properties of gNa0 (TTX-S, mostly Nav1.7) and gNa1 (TTX-R, mostly Nav1.8) conductances established through dynamic current-clamp simulation. The rightward shift in gNa1 activation compared to gNa0 indicates that TTX-R channels activate at more depolarized potentials, supporting sequential recruitment during action potential depolarization. Conductance normalized to 0-1. X-axis: holding potential (mV); Y-axis: normalized Na⁺ conductance. Curves: **Black** - gNa0 activation; **Red** - gNa0 inactivation; **Green** - gNa1 activation; **Blue** - gNa1 inactivation.

**Figure S6**


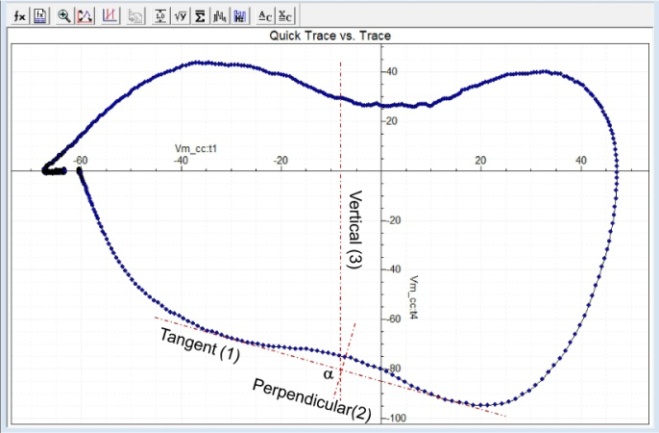

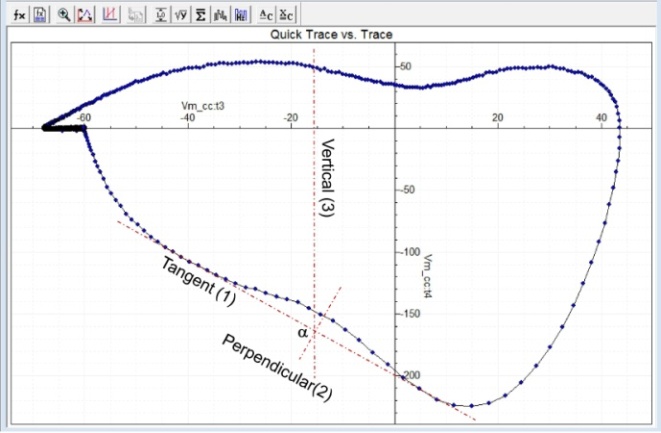


**Figure S6 Geometric method for determining TTX-R kick-in voltage from displacement current phase plots**. Representative displacement current phase plots from two C-type neurons (left and right panels) from sliced preparations, demonstrating the three-step geometric method for determining the kick-in voltage where TTX-R channels begin contributing to action potential depolarization. Method: (1) Draw tangent line (line #1) along the downward phase with visible inflection; (2) Draw perpendicular line (line #2) at the most prominent inflection point, creating intersection point (α) with the tangent line; (3) Draw vertical line (line #3) through point (α); the intersection with the horizontal axis indicates the kick-in voltage. This approach objectively identifies the voltage at which TTX-R channels begin contributing to total Na⁺ current during AP depolarization (Methods section 2.10). Axes: X-axis - membrane potential (mV); Y-axis - voltage derivative (mV/ms). Lines: Tangent (1), Perpendicular (2), Vertical (3) - dashed lines; α - intersection point.

**Figure S7**

**
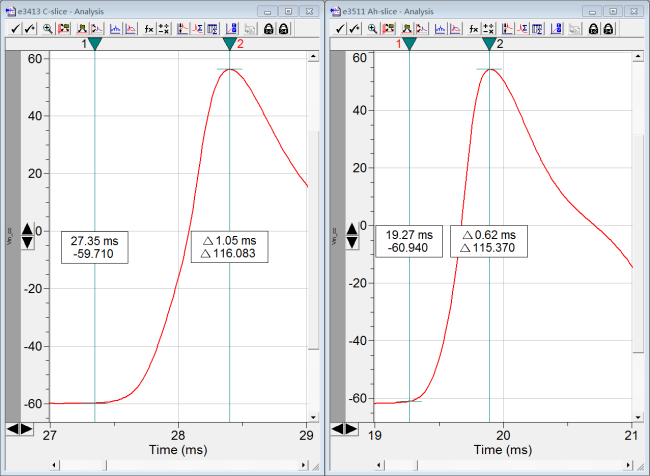

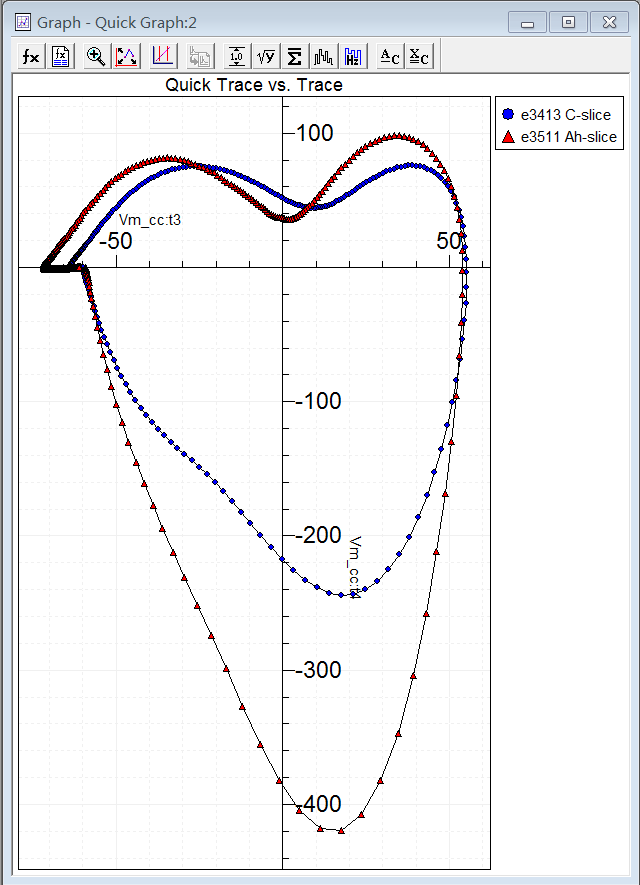
**

**Figure S7 Time-expanded action potential upstrokes from C-type and Ah-type neurons.** Expanded time-scale view of action potential upstrokes from sliced preparations. **Left**: C-type neuron. **Middle**: Ah-type neuron. **Right**: Overlaid comparison (blue - C-type; red triangles - Ah-type). Even with expanded temporal resolution, AP trajectories show smooth upstrokes without visible indication of dual TTX-S and TTX-R Na⁺ component activation. Annotations indicate voltage measurements at specific time points. This limitation demonstrates the necessity of voltage derivative and displacement current phase plot methods (Figures 1b-c, SF. 8) to resolve sequential Na⁺ channel recruitment. Axes: membrane potential (mV) vs. time (ms).

**Figure S8**

^
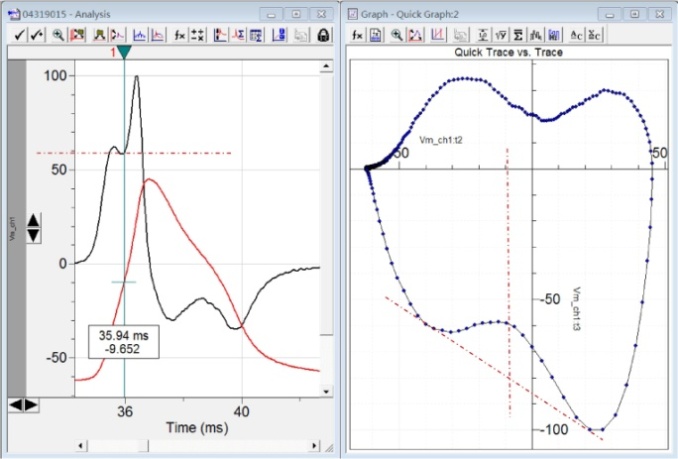

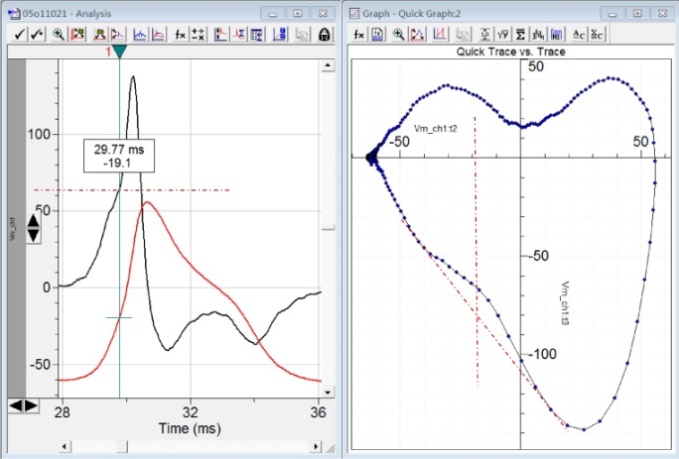
^

**Figure S8 Representative C-type APs with kick-in voltage measurements**. Two representative C-type neurons from sliced preparations showing measurement of TTX-R kick-in voltages. **Left panels**: AP trajectories (red) and voltage derivatives (black). **Right panels**: Displacement current phase plots. Line annotations: Horizontal dashed line - voltage derivative amplitude at inflection; vertical dashed line - kick-in voltage at the horizontal coordinate. These examples supplement Figure 1c, demonstrating sequential recruitment of TTX-R following TTX-S. Axes: Left panels - membrane potential (mV) and voltage derivative (mV/ms) vs. time (ms); Right panels - voltage derivative (mV/ms) vs. membrane potential (mV).

**Figure S9**


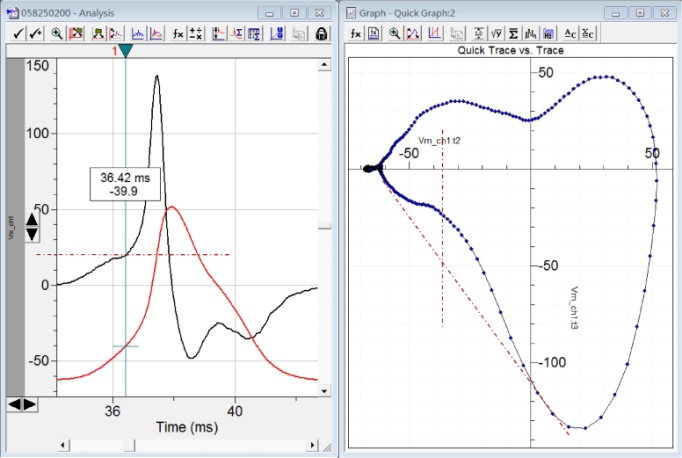

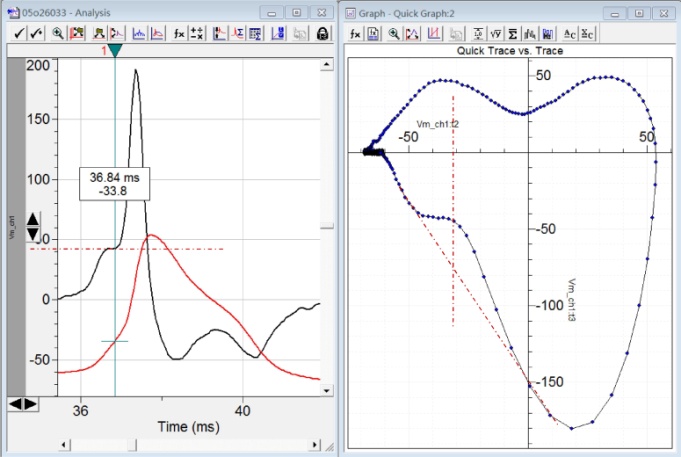


**Figure S9 C-type APs with unusually small TTX-S components.** Two examples of C-type neurons from large-scale screening of sliced preparations. In these atypical cases, the first Na⁺ component (TTX-S) was significantly smaller with more negative kick-in voltages (approximately -39.9 mV and -33.8 mV) compared to typical recordings in the main dataset. These recordings were excluded from the kick-in voltage analysis. **Left panels**: AP trajectories (red) and voltage derivatives (black). **Right panels**: Displacement current phase plots. **Line annotations**: Horizontal dashed line - voltage derivative amplitude at inflection; vertical dashed line - kick-in voltage at the horizontal coordinate. Axes: Left panels - membrane potential (mV) and voltage derivative (mV/ms) vs. time (ms); Right panels - voltage derivative (mV/ms) vs. membrane potential (mV).

**Figure S10**


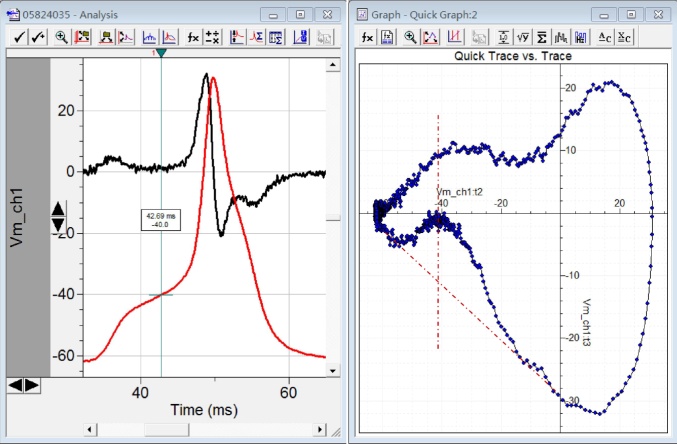


**Figure S10 Rare C-type AP with minimal TTX-S expression**. A rare C-type neuron from sliced preparation meeting all classification criteria (conduction velocity <1.0 m/s and characteristic waveform). However, this neuron exhibited an exceptionally small first Na^+^ component with voltage derivative <10 mV/ms (approximately 5 mV/ms). **Left panel**: AP trajectory (red) and voltage derivative (black) showing that the TTX-R component was recruited just before the first component returned to baseline. **Right panel**: Displacement current phase plot. The kick-in voltage was approximately -40 mV, substantially more negative than typical C-type neurons (see SF. 8, Figure 2). This extreme case suggests that when TTX-S expression is minimal, the measured kick-in voltage may approach the true activation threshold for TTX-R channels. Excluded from main dataset analysis due to atypical TTX-S expression. **Line annotations**: Horizontal dashed line - voltage derivative at inflection; vertical dashed line - kick-in voltage. Axes: Left panel - membrane potential (mV) and voltage derivative (mV/ms) vs. time (ms); Right panel - voltage derivative (mV/ms) vs. membrane potential (mV).

**Figure S11**


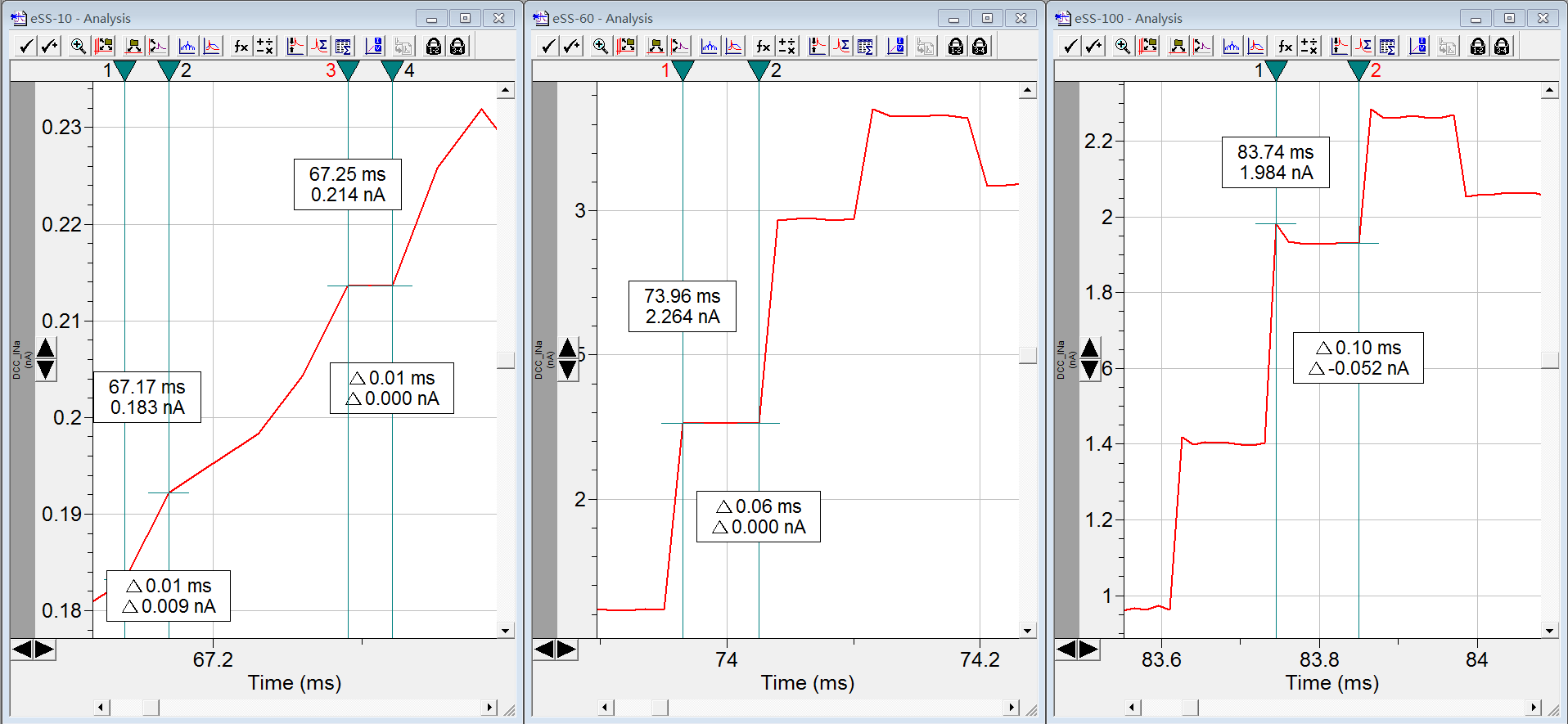


**Figure S11 Variation in gNa1 current injection with different step sizes.**

Representative gNa1 current injections from dynamic current-clamp with different integrated step sizes. **Left to right**: 10 μs, 60 μs, and 100 μs. Total gNa1 amplitude is identical across conditions; only the integrated step size varies. Increasing step size delays and broadens the current profile, affecting TTX-R recruitment dynamics during action potential generation (see Figures 5-6). **Axes**: Time (ms) vs. Current (nA).

**Figure S12**


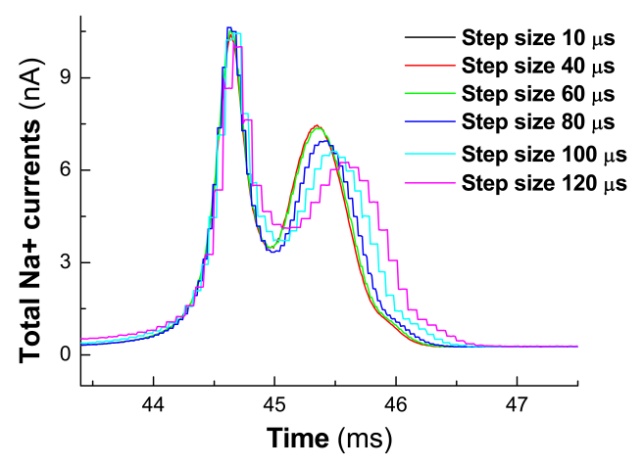


**Figure S12** **Representative recordings of total Na^+^**  **currents from DCC inputs with different step sizes.** Total Na^+^ currents from DCC inputs showing the first peak (gNa0/TTX-S) and second peak (gNa1/TTX-R) with different integrated step sizes ranging from 10 to 120 μs. Color-coded traces: Black - 10 μs; Red - 40 μs; Green - 60 μs; Blue - 80 μs; Pink - 100 μs; Orange - 120 μs. The first peak remains relatively stable across conditions, while the second peak progressively decreases and shifts rightward with increasing step size, demonstrating differential sensitivity of TTX-R recruitment to temporal integration parameters. This supports the analysis in Figure 6c. Axes: Time (ms) vs. Total Na^+^ currents (nA).
